# Evaluation of the acute toxicity of the extracts of *Anacyclus pyrethrum* var. *pyrethrum* (L.) and *Anacyclus pyrethrum* var. *depressus* Maire in Swiss mice

**DOI:** 10.14202/vetworld.2021.457-467

**Published:** 2021-02-22

**Authors:** Fatima Zahra Jawhari, Abdelfattah El Moussaoui, Hamada Imtara, Hamza Mechchate, Imane Es-Safi, Mohamed Bouhrim, Loubna Kharchoufa, Achraf Miry, Dalila Bousta, Amina Bari

**Affiliations:** 1 Laboratory of Biotechnology, Environment, Agri-Food, and Health LBEAS, Faculty of Sciences, University Sidi Mohamed Ben Abdellah USMBA Fez, Morocco; 2 Faculty of Arts and Sciences, Arab American University Palestine, P. O. Box 240, Jenin, Palestine; 3 Laboratory of Bioresources, Biotechnology, Ethnopharmacology and Health, Faculty of Sciences, Mohammed First University, Oujda, Morocco; 4 Laboratory of University Hospital Mohammed 6 Oujda, Morocco

**Keywords:** acute oral toxicity, *Anacyclus pyrethrum* var. *depressus* (Ball) Maire, *Anacyclus pyrethrum* var. *pyrethrum* (L), histopathology, serum biochemistry analysis

## Abstract

**Background and Aim:**

*Anacyclus pyrethrum* (L.) has been used in traditional North African and Indian medicine for the treatment of several diseases such as cancer, rheumatism, epilepsy, diabetes, and Alzheimer’s disease. Despite its medical benefits, few studies have examined its toxicity. The present study evaluated the acute toxicity of hydroethanolic extracts of different parts (roots, seeds, leaves, and capitula) of two varieties of *A. pyrethrum* (L.), namely, *A. pyrethrum* var. *pyrethrum* (L) and *A. pyrethrum* var. *depressus* (Ball) Maire, in mice.

**Materials and Methods:**

Acute toxicity was evaluated after the oral administration of different extracts at doses of 300, 500, and 2000 mg/kg. Mortality, body weight, general behavior, and adverse effects were observed daily for 14 days. At the end of the experiment, mice were sacrificed, and biochemical parameters and histopathology of the liver, kidneys, and spleen were analyzed.

**Results:**

The extracts of different parts of both plants induced no signs of toxicity or mortality during the observation period, excluding capitulum and seed extracts, which induced slight sedation at a dose of 2000 mg/kg. The LD_50_ of the extracts was estimated to exceed 2000 mg/kg. The administration of *A. pyrethrum* var. *pyrethrum* roots at a dose of 300 mg/kg resulted in significantly increased AST levels. However, the *A. pyrethrum* var. *depressus* root extract induced significant increases in the levels of both transaminases (alanine aminotransferase [ALT] and aspartate aminotransferase [AST]). The remaining extracts of both plants at a dose of 500 mg/kg significantly increased AST levels. Moreover, all plant extracts excluding the *A. pyrethrum* var. *pyrethrum* capitulum extract at 2000 mg/kg provoked significant increases in AST levels, and *A. pyrethrum* var. *depressus* roots provoked a significant increase of ALT levels. Meanwhile, mice treated with high doses of extracts (2000 mg/kg) displayed histopathological changes in the liver, kidneys, and spleen characterized by hepatic distress, inflammatory infiltration, focal tubular necrosis, vascular congestion, and lymphoid hyperplasia.

**Conclusion:**

The results of the present study indicate that the hydroethanolic extracts of different parts of two varieties of *A. pyrethrum* (L.) were not toxic in mice at low concentrations, whereas some toxic effects were detected in mice treated at 2000 mg/kg.

## Introduction

The use of medicinal plants, whether as pure compounds, extracts, or derivatives, is a real treasure for humanity because they produce a wide range of drugs for treating various illnesses. *Anacyclus pyrethrum* (L.) is a plant that belongs to the Asteraceae family and genus *Anacyclus* [1]. It is commonly known as pellitory, Aqar Qarha, Oud El Attas, African pyrethrum, and Akarkara. It is endemic to Morocco, Algeria, and Spain, and it has been introduced in India, Australia, France, Germany, Nepal, Pakistan, Poland, and Ukraine. In traditional medicine, the roots of this plant are recommended as a sialagogue for treating several diseases and ailments. They are used in the form of a mouthwash to treat toothache and problems related to saliva secretion, and they are used as a gargle to treat angina, digestion problems, paralysis of the tongue, and lethargy. Moreover, the decoction of the roots is also used to treat paralysis of the limbs, and the roots can also be used in the form of cream based on animal fats to treat gout and sciatica [2,3]. Other pharmacological and biological properties have been reported in the literature, such as antibacterial and antifungal activities [4-6], aphrodisiac properties [7-9], effects against gout and sciatica [10], immunostimulant properties [11-14], anti-convulsive activity [7,15,16], antidiabetic effects [17,18], anti-parasitic and antibiotic activities [19], insecticidal properties [20], antioxidant effects [14,21], anti-inflammatory properties [22], local anesthetic effects [23], anti-epileptic properties [15,24], memory-enhancing effects [25], anti-rheumatic activity [20], and wound-healing properties [26]. These properties are the results of the phytochemical constituents of the plant, such as phenolic compounds, flavonoids, alkaloids, and tannins[22].

In recent decades, much research has been conducted on the therapeutic properties of many medicinal plants, but there is little information concerning their toxic or harmful effects [27]. In addition, no study has described the toxicity profile or side effects of different parts of *A. pyrethrum* (L.). In Morocco, two varieties of *A. pyrethrum* (L.) have been identified: *A. pyrethrum* var. *pyrethrum* (L.) and *A. pyrethrum* var. *depressus* (Ball) Maire [28,29].

Thus, the present study was conducted to evaluate the acute toxicity of different extracts (roots, seeds, leaves, and capitula) of *A. pyrethrum* var. *pyrethrum* (L.) and *A. pyrethrum* var. *depressus* (Ball) Maire in Swiss albino mice.

## Materials and Methods

### Ethical approval

The ethical institutional committee, Faculty of Sciences Dhar El Mahraz, University Sidi Mohamed Ben Abdallah, Fez, Morocco, approved the protocol. All the experimental proceedings achieved in laboratory animals followed the internationally accepted standard guidelines for animal care. The authors tried to minimize animal suffering and the number of animals used.

### Study period and location

This study was conducted in Septembre 2019 at Sidi Mohamed Ben Abdellah University, Morocco.

### Collection and extraction of plant material

The two varieties of *A. pyrethrum* (L.) were collected in July 2019 from the Timahdite region in the Middle Atlas, Morocco. Then, the plants were identified and authenticated by Professor Bari Amina, a botanist in the Department of Biology, Laboratory of Biotechnology, Environment, Agri-Food, and Health, Faculty of Sciences, University Sidi Mohamed Ben Abdellah (USMBA) Fez, Morocco. These specimens have been deposed in the herbarium under the voucher numbers A31/31-5-18/TM and A32/31-5-18/TM.

### Preparation of plant extracts

Different parts of both plants were washed with tap water, separated, and dried in the shade for 1 week at room temperature. Then, the parts were crushed and reduced to a coarse powder. Then, 10 g of each powder were cold-macerated with ethanol (70%) for 48 h, the extracts obtained were filtered and concentrated under reduced pressure at 40°C, and the obtained residues were stored at 4°C until use [30].

### Experimental animals

One hundred twenty-five male Swiss mice (30±2 g) were used. The animals were housed in Makrolon cages under standard laboratory conditions (12 h light/12 h darkness, 21±2°C, relative humidity=55±5%). The animals were given a standard pellet diet and water *ad libitum* throughout the experimental period.

### Acute toxicity study

The acute toxicity study was conducted following OECD guideline No. 425 (OECD, 2008). The animals were randomly divided into 25 groups (five mice per group). The treated animals received the plant extracts at a dose of 300, 500, or 2000 mg/kg, and the control group received distilled water (10 ml/kg). After a single dose of each plant extract was administered, the animals were followed for 4 h each day for 14 days to note changes in behavior and signs of toxicity (digestion, body weight, food intake, urination, skin changes, sedation, diarrhea, and death). At the end of the experiment (2 weeks), the animals were subjected to light diethyl ether anesthesia, and blood was obtained through cardiac puncture. Next, the animals were sacrificed. Then, plasma was separated by centrifugation at 3000 rpm at 4°C for 20 min to assess biochemical parameters, and organ tissue (liver, kidneys, and spleen) was conserved to conduct histological examinations.

The relative organ weight (ROW) was calculated according to the following formula [31]:

O_w_: organ weight

B_w_: body weight.

### Biochemical parameters

The biochemical parameters were measured in plasma. Urea levels were determined using the enzymatic method [32], creatinine levels were measured using the Jaffe method [33], and ALT and AST levels were measured using the IFCC method without pyridoxal-5-phosphate [34]. All tests were performed with the Architect c8000 automated analyzer using commercial reagent kits.

### Histological analysis

The organ tissues of the animals used in this study were prepared for the examination of microscopic lesions. A 10% buffered formalin solution was used to secure the organs within 48 h, and the organs were embedded in paraffin. The samples were cut to a thickness of approximately 4-5 μm from tissue sections using a rotating microtome, and the mounted glass blades were kept on a heating plate (54°C) overnight [35]. Finally, the protocol for hematoxylin and eosin staining was used.

### Statistical analysis

The analysis was performed using Graph Prism version 7 software (San Diego, California, USA), statistical processing was performed through analysis of variance, and comparisons of means were made using the Tukey test. The data were expressed as the mean±SEM.

## Results

### Behavioral parameter assessment

During the experiment, no mortalities were recorded. However, the mice treated with capitulum and seed extracts of both plants (APPC, APPG, APDC, and APDG) at a dose of 2000 mg/kg displayed slight signs of sedation during the first 4 h after oral administration (Table-1). Therefore, the LD_50_ of the different extracts both varieties studied exceeded 2000 mg/kg.

**Table-1:** The behavioral observations of acute toxicity in mice.

	Samples code	Doses mg/kg	Observation							

			Digestion	Body weight	Food intake	Urination	Change in skin	Sedation	Diarrhea	Death
*Anacyclus Pyrethrum* var. *Pyrethrum*	Roots (APPR)	300	−	−	−	−	−	−	−	−
		500	−	−	−	−	−	−	−	−
		2000	−	−	−	−	−	−	−	−
	Seeds (APPG)	300	−	−	−	−	−	−	−	−
		500	−	−	−	−	−	−	−	−
		2000	−	−	−	−	−	+	−	−
	Leaves (APPF)	300	−	−	−	−	−	−	−	−
		500	−	−	−	−	−	−	−	−
		2000	−	−	−	−	−	−	−	−
	Capitulas (APPC)	300	−	−	−	−	−	−	−	−
		500	−	−	−	−	−	−	−	−
		2000	−	−	−	−	−	+	−	−
*Anacyclus Pyrethrum* var. *Depressus*	Roots (APDR)	300	−	−	−	−	−	−	−	−
		500	−	−	−	−	−	−	−	−
		2000	−	−	−	−	−	−	−	−
	Seeds (APDG)	300	−	−	−	−	−	−	−	−
		500	−	−	−	−	−	−	−	−
		2000	−	−	−	−	−	+	−	−
	Leaves (APDF)	300	−	−	−	−	−	−	−	−
		500	−	−	−	−	−	−	−	−
		2000	−	−	−	−	−	−	−	
	Capitulas (APDC)	300	−	−	−	−	−	−	−	−
		500	−	−	−	−	−	−	−	−
		2000	−	−	−	−	−	+	−	−
	Control	Physiological water	−	−	−	−	−	−	−	−

−=Not observed; +=Observed. APPR=*Anacyclus pyrethrum* var. *pyrethrum* roots, APPG=*Anacyclus pyrethrum* var. *pyrethrum* seeds, APPF=*Anacyclus pyrethrum* var. *pyrethrum* leaves, APPC=*Anacyclus pyrethrum* var. *pyrethrum* capitula, APDR=*Anacyclus pyrethrum* var. *depressus* roots, APDG=*Anacyclus pyrethrum* var. *depressus* seeds, APDF=*Anacyclus pyrethrum* var. *depressus* leaves, APDC=*Anacyclus pyrethrum* var. *depressus* capitula

### Body weight

**Figure-1:**
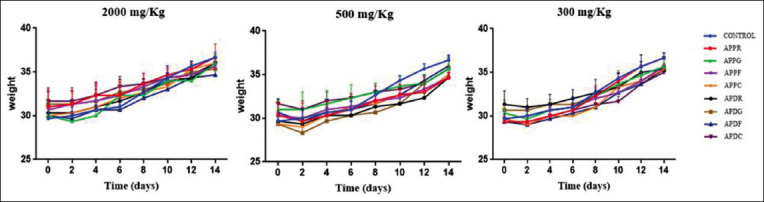
Body weight of mice in the treated and control groups during the 14-day acute toxicity assessment.

According to Figure-1, the different plant extracts did not induce any abnormal changes in the body weight of the mice. Moreover, there was no significant difference in the changes of body weight between the control group and the treatment groups, which proves the absence of toxicity.

### ROW

The results of the effect of the plant extracts on organ weight are presented in Table-2. There was no significant difference in ROW or the general appearance of organs (liver, spleen, and kidneys) between the treatment groups and the control group.

**Table-2:** Relative organ weights of mice, 14 days after acute oral administration of extracts from different parts of the two varieties *Anacyclus pyrethrum* var. *pyrethrum* (L) and *Anacyclus pyrethrum var. depressus* (Ball) Maire.

	Samples code	Doses mg/kg	Relative organ weights		

			liver	kidneys	spleen
*Anacyclus Pyrethrum* var. *Pyrethrum*	Roots (APPR)	300	6.53±0.46	1.30±0.14	0.86±0.04
		500	7.16±0.31	1.25±0.11	0.91±0.06
		2000	6.36±0.63	1.25±0.08	0.58±0.05
	Seeds (APPG)	300	6.25±0.50	1.30±0.05	0.77±0.1
		500	6.78±0.02	1.25±0.11	0.69±0.39
		2000	5.5±1.34	1.02±0.15	0.81±0.14
	Leaves (APPF)	300	6.56±0.19	1.27±0.09	0.81±0.03
		500	6.63±0.49	1.36±0.11	0.80±0.08
		2000	5.80±0.09	1.49±0.21	0.67±0.10
	Capitulas (APPC)	300	6.72±0.6	1.36±0.07	0.79±0.14
		500	6.39±0.33	1.33±0.13	0.73±0.23
		2000	5.28±0.64	1.27±0.22	0.69±0.09
*Anacyclus Pyrethrum* var. *Depressus*	Roots (APDR)	300	7.15±0.03	1.32±0.07	0.81±0.01
		500	6.60±0.28	1.32±0.05	0.84±0.01
		2000	7.85±0.28	1.42±0.11	0.95±0.2
		300	6.51±0.67	1.24±0.03	0.78±0.06
	Seeds (APDG)	500	6.5±0.14	1.37±0.11	0.76±0.02
		2000	6.41±0.59	1.16±0.08	1.11±0.39
	Leaves (APDF)	300	6.19±0.05	1.29±0.07	0.78±0.04
		500	7.09±0.94	1.24±0.08	0.70±0.06
		2000	7.05±0.56	1.25±0.06	1.260.18
	Capitulas (APDC)	300	6.76±0.28	1.31±0.06	0.82±0.14
		500	6.35±1.06	1.35±0.11	0.75±0.14
		2000	7.33±0.16	1.59±0.15	0.71±0.02
	Control	physiological water	6.34±0.31	1.31±0.13	0.79±0.13

### Biochemical parameters

The effect of the plant extracts on AST, ALT, creatinine, and urea levels is presented in Table-3. The root extracts of both plants (APPR and APDR) at a dose of 300 mg/kg significantly increased AST levels, but no significant changes in ALT, urea, and creatinine levels were noted. A dose of 500 mg/kg induced a significant increase in AST level for the root, seed, and capitulum extracts of both plants, and no significant change was observed in the other biochemical parameters. However, a dose of 2000 mg/kg provoked a significant increase in AST levels for all plant extracts excluding the capitulum extract of *A. pyrethrum* var. *pyrethrum* plant (APPC). Moreover, at the same dose, the root extract of *A. pyrethrum* var. (APDR) provoked a significant increase in ALT levels in comparison with the control group levels, whereas no significant changes were observed for the other biochemical parameters.

**Table-3:** Biochemical parameters of mice serum after acute oral administration of extracts of the different parts of the two varieties studied.

	Simples code	Doses mg/kg	Biochemical parameters			

			AST	ALT	Urea	Creatinine
*Anacyclus Pyrethrum* var. *Pyrethrum*	Roots (APPR)	300	250±6.65***	30.33±1.33	0.28±0.007	4.23±0.14
		500	261±4.05**	31±2.02	0.38±0.012	4±0.208
		2000	315±14**	54±4.04	0.37±0.02	4.5±0.28
	Seeds (APPG)	300	285.66±8.17	32±2.9	0.37±0.01	3.5±0.28
		500	257.7±4.05**	40.35±3.52	0.3±0.03	3.56±0.29
		2000	210±9.07***	50.7±1.85	0.40±0.06	4.23±0.62
	Leaves (APPF)	300	277±4.93	36.6±4.09	0.29±0.04	3.93±0.43
		500	275±4.63	30±0.57	0.35±0.01	3.46±0.08
		2000	232±5.92***	39±3.17	0.40±0.04	3.54±0.29
	Capitulas (APPC)	300	286.33±8.08	42.53±2.02	0.38±0.01	3.73±0.17
		500	258.7±3.28**	36±1.52	0.31±0.03	3.63±0.27
		2000	296±6.48	32±6.11	0.32±0.01	3.5±0.28
*Anacyclus Pyrethrum* var. *Depressus*	Roots (APDR)	300	255±5.78**	31.55±3.71	0.28±0.17	4±0.57
		500	254.3±3.52**	32±2.08	0.42±0.01	3.43±0.14
		2000	326±15.37***	63±7.21*	0.48±0.05	4.23±0.39
	Seeds (APDG)	300	285±9.84	41.66±1.76	0.31±0.01	4.06±0.12
		500	258±2.64**	33.8±2.4	0.28±0.03	4±0.12
		2000	234±12.41***	26.4±3,71	0.44±0.02	3.4±0.33
	Leaves (APDF)	300	281.3±8.76	30.33±3.52	0.30±0.03	4.33±0.33
		500	272±4.37	31±0,88	0.31±0.02	3.36±0.20
		2000	254.7±10.89*	50.6±8.29	0.42±0.04	3.96±0.54
	Capitulas (APDC)	300	275.23±13.1	35±2.02	0.41±0.009	3±0.23
		500	260±4.80**	35.37±5.04	0.29±0.017	3.53±0.29
		2000	235±11.71***	48±6.55	0.52±0.08	5±0.57
	Control	Physiological water	278±4.35	39±1.73	0.30±0.01	3.6±0.32

Values are expressed mean±SEM. AST=Aspartate aminotransferase, ALT=Alanine aminotransferase.

* Correlation is significant at the level *P<*0.05.

** Correlation is significant at the level *P<*0.01

*** Correlation is significant at the level *P<*0.001. Values are expressed mean±SEM. APPR=*Anacyclus pyrethrum* var. *pyrethrum* roots, APPG=*Anacyclus pyrethrum* var. *pyrethrum* seeds, APPF=*Anacyclus pyrethrum* var. *pyrethrum* leaves, APPC=*Anacyclus pyrethrum* var. *pyrethrum* capitula, APDR=*Anacyclus pyrethrum* var. *depressus* roots, APDG=*Anacyclus pyrethrum* var. *depressus* seeds, APDF=*Anacyclus pyrethrum* var. *depressus* leaves, APDC=*Anacyclus pyrethrum* var. *depressus* capitula

### Histopathological analysis

**Figure-2:**
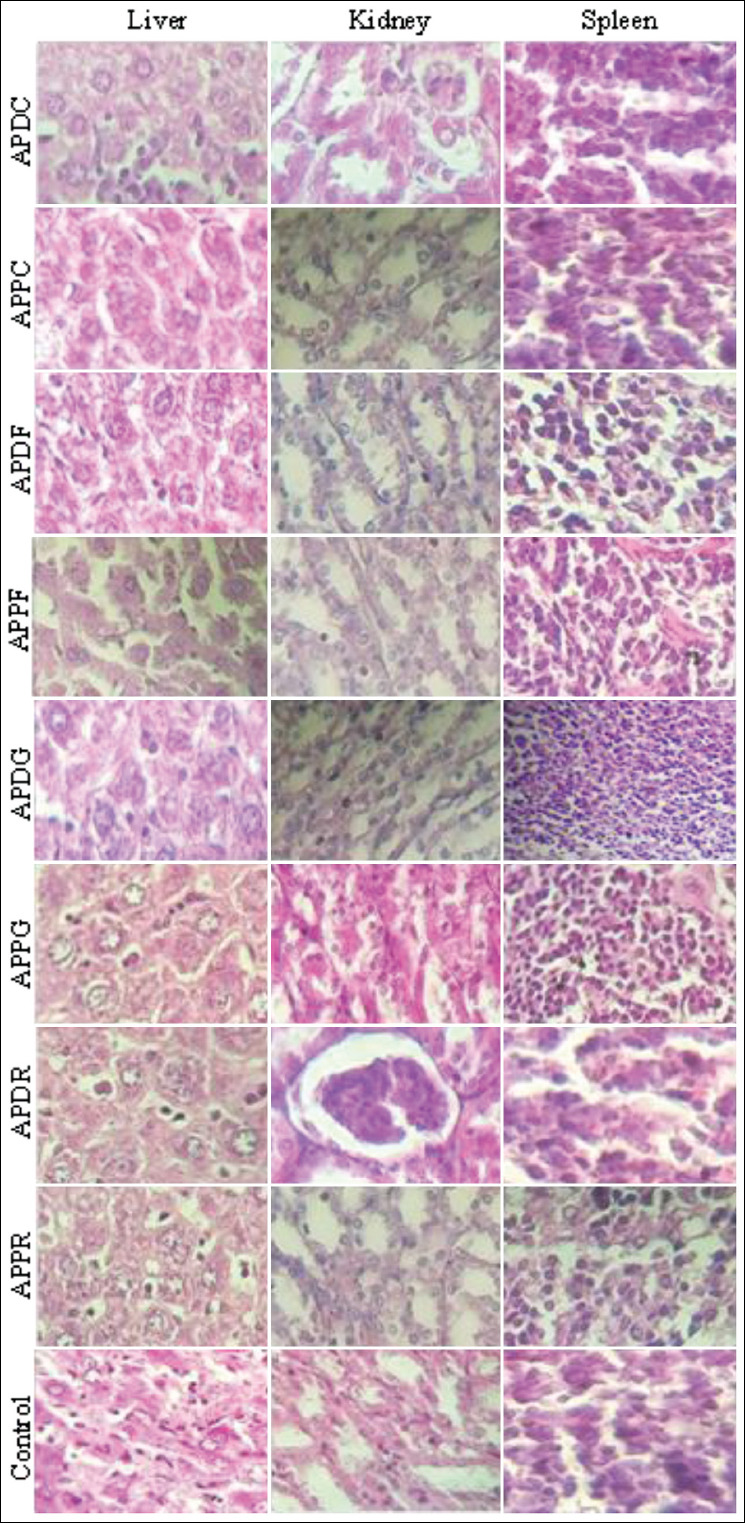
Histopathological observation of mice organs (liver, kidney, and spleen) from the control and the groups treated with 300 mg/Kg of the extracts from the different parts of the two varieties *Anacyclus pyrethrum* var. *pyrethrum* (L) and *Anacycluspyrethrum* var. *depressus* (Ball) Maire.

**Figure-3:**
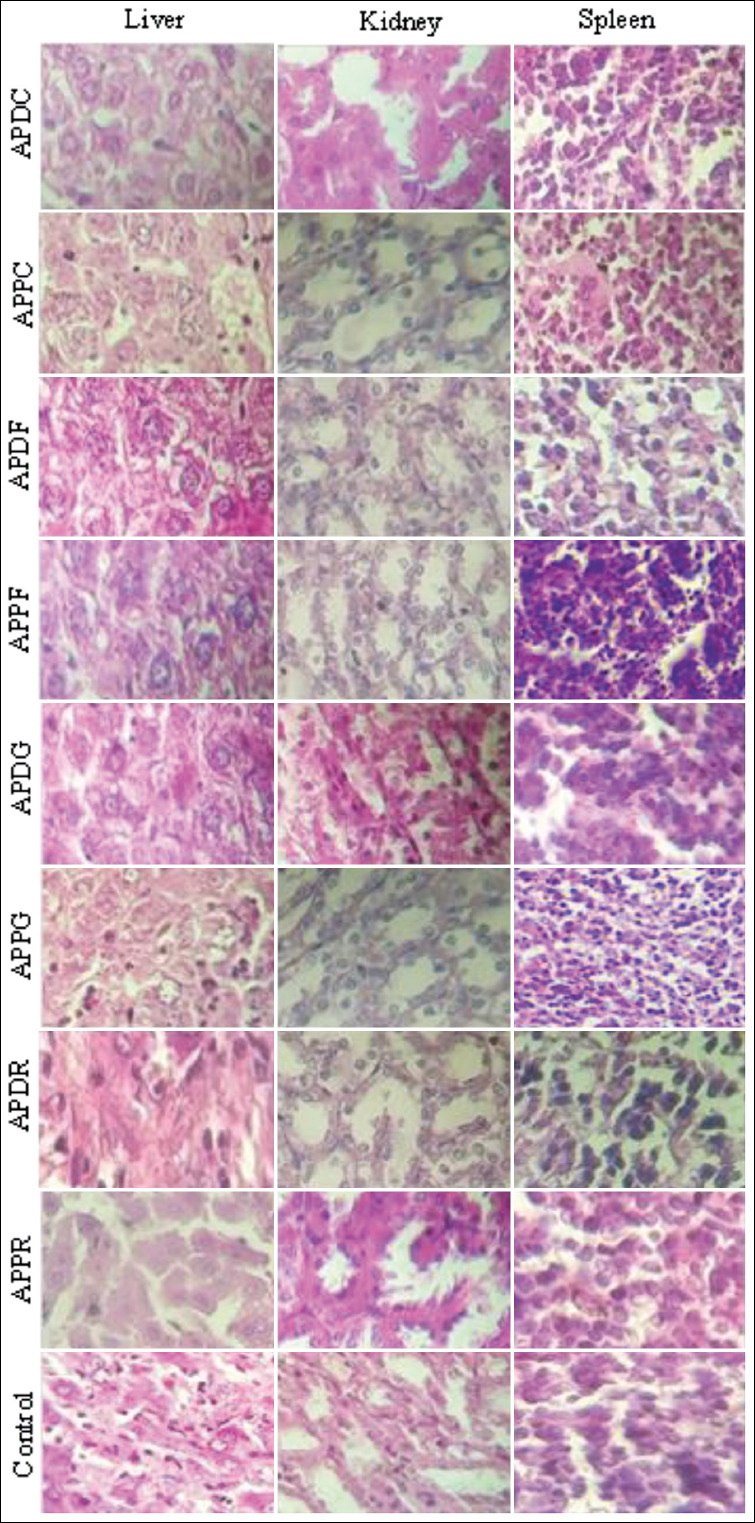
Histopathological observation of mice organs (liver, kidney, and spleen) from the control and the groups treated with 500 mg/Kg of the extracts from the different parts of the two varieties *Anacyclus pyrethrum* var. *pyrethrum* (L) and *Anacyclus pyrethrum* var. *depressus* (Ball) Maire.

**Figure-4:**
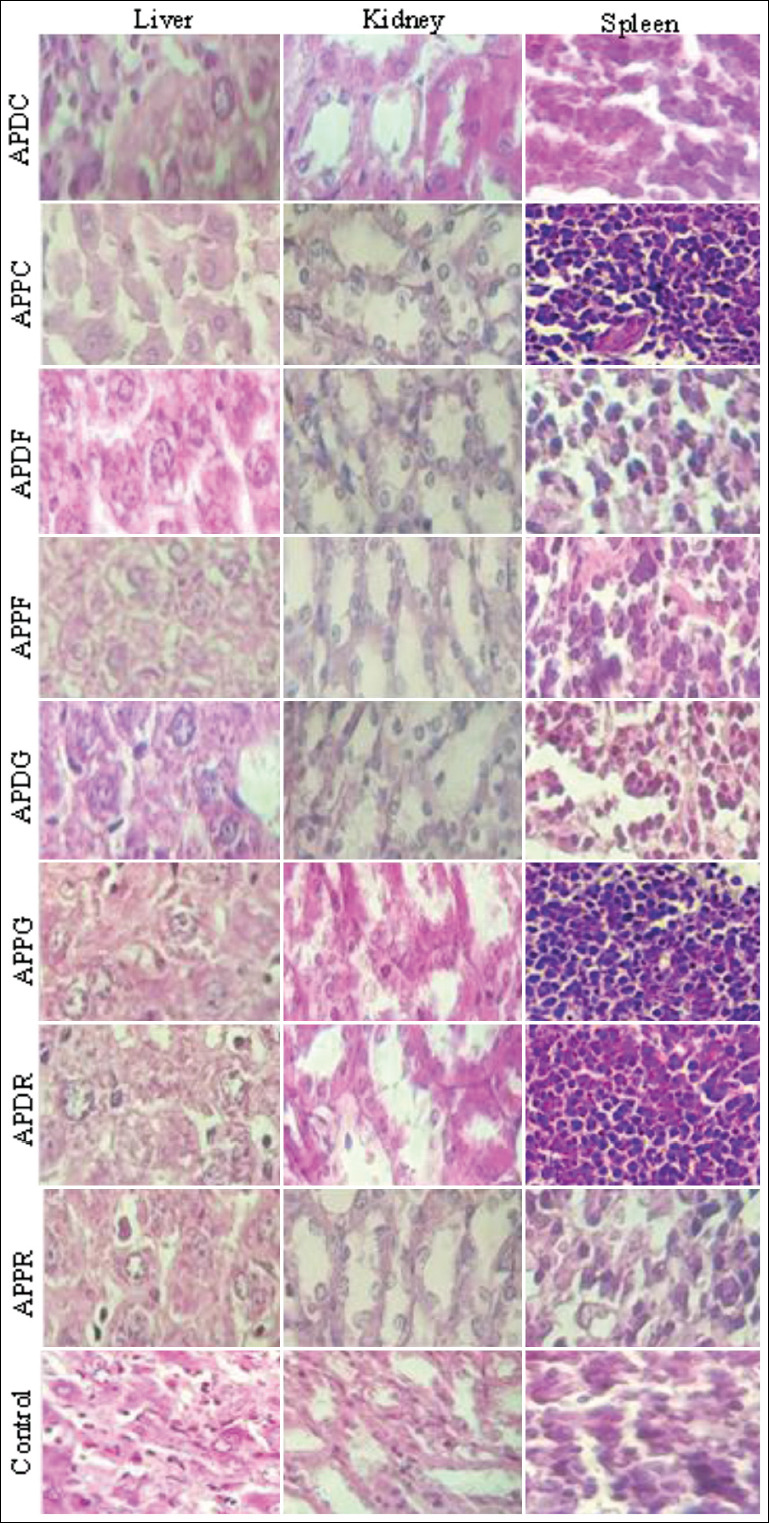
Histopathological observation of mice organs (liver, kidney, and spleen) from the control and the groups treated with 2000 mg/Kg of the extracts from the different parts of the two varieties *Anacyclus pyrethrum* var. *pyrethrum* (L) and *Anacyclus pyrethrum* var. *depressus* (Ball) Maire.

The results of the histological examination for the liver, spleen, and kidneys in the treatment and control groups are presented in Figures-2-4, respectively. Microscopic examination of all tissue sections collected (liver, spleen, and kidneys) from mice administered extracts of both plants at a dose of 300 mg/kg exhibited a normal architecture and no abnormalities, alterations, or degenerative or infiltrative lesions (Table-4). The leaf extracts (APPF and APDF) of both plants at a dose of 500 mg/kg induced hepatocyte damage, whereas sinusoid dilatation was observed in the mice treated with the capitulum extract of *A. pyrethrum* var. *pyrethrum* (APPC). However, no abnormalities were noted in the spleen and kidneys. Meanwhile, the doses of 500 and 2000 mg/kg induced hepatocyte damage in mice treated with the leaf and seed extracts of both plants (ADPF, APPF, APDG, and APPG), whereas sinusoid dilatation observed in the livers of mice treated with *A. pyrethrum* var. *pyrethrum* (APPC). Inflammatory infiltration of the portal and lobal spaces was observed in the livers of mice treated with the roots extract of *A. pyrethrum* var. *depressus* (APDR). Moreover, focal tubular necrosis was observed in the kidneys of mice treated with the roots, seeds, leaves, and capitula of *A. pyrethrum* var. *pyrethrum*. However, vascular congestion and lymphoid hyperplasia were observed in the spleens of mice treated with *A. pyrethrum* var. *pyrethrum* extracts excluding the root extract (APPC).

**Table-4:** Histopathological observation of mice organs (liver, kidney, and spleen) from the groups treated with 300, 500, and 2000 mg/Kg of the extracts from the different parts of the two varieties *Anacyclus pyrethrum* var. *pyrethrum* (L) and *Anacyclus pyrethrum* var. *depressus* (Ball). Maire.

	Samples code	Doses mg/kg	Organs		

			liver	kidneys	spleen
*Anacyclus Pyrethrum* var. *Pyrethrum*	Roots (APPR)	300	-	-	-
		500	-	-	-
		2000	-	Focal tubular necrosis	-
	Seeds (APPG)	300	-	-	-
		500	-	-	-
		2000	Hepatocyte suffering	Focal tubular necrosis	Lymphoid hyperplasia
	Leaves (AwPPF)	300	-	-	-
		500	Hepatocyte suffering	-	-
		2000	Hepatocyte suffering	Focal tubular necrosis	Vascular congestion Lymphoid hyperplasia
	Capitulas (APPC)	300	-	-	-
		500	Sinusoid Dilatation	-	-
		2000	Sinusoid Dilatation	Focal tubular necrosis	Lymphoid hyperplasia
*Anacyclus Pyrethrum* var. *Depressus*	Roots (APDR)	300	-	-	-
		500	-	-	-
		2000	Inflammatory infiltration	-	Vascular congestion
	Seeds (APDG)	300	-	-	-
		500	-	-	-
		2000	Hepatocyte suffering	-	Lymphoid hyperplasia
	Leaves (APDF)	300	-	-	-
		500	Hepatocyte suffering	-	-
		2000	Hepatocyte suffering	-	Vascular congestion Lymphoid hyperplasia
	Capitulas (APDC)	300	-	-	-
		500	-	-	-
		2000	Glycogenic nuclei	-	Lymphoid hyperplasia Vascular congestion

APPR=*Anacyclus pyrethrum* var. *pyrethrum* roots, APPG=*Anacyclus pyrethrum* var. *pyrethrum* seeds, APPF=*Anacyclus pyrethrum* var. *pyrethrum* leaves, APPC=*Anacyclus pyrethrum* var. *pyrethrum* capitula, APDR=*Anacyclus pyrethrum* var. *depressus* roots, APDG=*Anacyclus pyrethrum* var. *depressus* seeds, APDF=*Anacyclus pyrethrum* var. *depressus* leaves, APDC=*Anacyclus pyrethrum* var. *depressus* capitula

## Discussion

The toxicity of a plant is defined as the potential of its extracts to cause harm to humans or animals. These effects can include harm to cells, organs, or the whole body. The symptoms caused by the toxic effects of plants are dominated by the following areas: Brain (e.g., convulsions, paralysis, depression, anorexia, shivers, dizziness, and coma), gastrointestinal tract (nausea, vomiting, and diarrhea), kidneys (acute renal failure, urination, and necrosis), and liver (liver dysfunction and cell destruction, necrosis, and lobar hepatitis) [36]. The results of this study demonstrated that the intake of these plant extracts did not induce the appearance of toxic symptoms, excluding the capitulum and seed extracts of both species at a dose of 2000 mg/kg. At this dose, mice exhibit mild signs of sedation during the first 4 h after oral ­administration of the extracts, and this symptom is explained by the fact that the plant has sedative and anesthetic effects [23]. The administration of the extracts of these two plants did not cause death in the mice. It can be deduced from this finding that the LD_50_ of the extracts of the different parts of the two plant species is >2000 mg/kg, and these results are in agreement with other studies [37,38].

The root extracts of both plant at a dose of 300 mg/kg induced a significant increase of AST levels but no significant variation of ALT levels. Serum ALT and AST levels can highlight structural damage in the liver and help in the diagnosis of liver disease [39]. They have high diagnostic value in hepatology. A high concentration of AST in serum indicates lesions in different parts of the body, but ALT is more specific for liver damage, specifically explaining liver dysfunction [40]. Regarding the effect of these extracts on liver function, transaminase levels (ALT and AST) were determined. Therefore, it can be concluded that the extracts that increased AST levels could be poisoning different parts of the body. However, the roots of *A. pyrethrum* var. *depressus* were hepatotoxic because they significantly increased the levels of both transaminases (ALT and AST). The administration of root, seed, and capitulum extracts of both plants at a dose of 500 mg/kg induced a significant increase in AST levels but no significant changes in the other biochemical parameters. However, all plants extracts excluding the *A. pyrethrum* var. *pyrethrum* capitulum extract at a dose of 2000 mg/kg provoked a significant increase in AST levels. Therefore, these extracts caused damage in different parts of the body. Moreover, creatinine and urea are considered the most important markers of renal functions [41]. They are products eliminated naturally by the kidneys, and high serum levels of these compounds represent an indication or sign of deterioration of kidney function [42]. From these results, we conclude that these extracts do not induce nephrotoxicity as long as they do not cause changes in the levels of renal biomarkers (urea and creatinine). Histopathological analysis of animal organs provides the basis for evaluating the safety of treatment [43]. The results of the histological examination of the liver, kidneys, and spleen revealed no abnormalities in these organs ­associated with the administration of the various extracts the low doses. However, the groups of mice treated at a high dose (2000 mg/kg) exhibited histopathological changes in the liver, kidneys, and spleen characterized by hepatic distress, inflammatory infiltration, focal tubular necrosis, vascular congestion, and lymphoid hyperplasia. These abnormalities in these vital organs could be attributable to the presence of high concentrations of alkaloids in the administered extracts [44].

## Conclusion

In the present work, the acute oral toxicity of the hydroethanolic extracts of different parts of two varieties of *A. pyrethrum* (L.) was examined in mice. The results for acute toxicity illustrated that extracts of all parts of both plants exerted some toxic effects in mice at a high dose (2000 mg/kg) as indicated by serum biochemical and/or histological analyses. However, subacute and chronic toxicity studies are required to evaluate the safe use of these plants in the long term.

It is important to note that this is the first study to provide data on the acute toxicity of extracts of different parts of *A. pyrethrum* var. *pyrethrum* (L) and *A. pyrethrum* var. *depressus* (Ball) Maire. This should be extremely useful for reference purposes and any future study of this herbal medicine.

## Data Availability

The data used to support the findings of this study are available from the corresponding author upon request.

## Authors’ Contributions

HI, DB, and AB: Conceptualization and supervision. FZJ, AE, IE, AM, and HM: Methodology, laboratory experiments, and biochemical analysis. FZJ, HI, MB, and LK: Manuscript writing. HI: Review of the draft. All authors read and approved the final manuscript.
